# Low blood levels of high-density lipoprotein (HDL) cholesterol are positively associated with cancer

**DOI:** 10.1007/s00432-021-03867-1

**Published:** 2021-12-12

**Authors:** Sven H. Loosen, Karel Kostev, Mark Luedde, Tom Luedde, Christoph Roderburg

**Affiliations:** 1 Clinic for Gastroenterology, Hepatology and Infectious Diseases, University Hospital Düsseldorf, Medical Faculty of Heinrich Heine University Düsseldorf, Moorenstraße 5, 40225 Düsseldorf, Germany; 2 Epidemiology, IQVIA, Frankfurt, Germany; 3 KGP Bremerhaven, Bremerhaven, Germany

**Keywords:** LDL, HDL, Triglycerides, Lipid metabolism, Malignancy

## Abstract

**Purpose:**

There is a growing body of evidence suggesting a decisive involvement of the human lipid metabolism in cancer development. However, clinical data on the association between blood triglyceride or cholesterol levels including the cholesterol transporters high-density and low-density lipoproteins (LDL, HDL) and cancer incidence have remained inconclusive. Here, we investigated the association between blood triglyceride as well as total, LDL and HDL cholesterol levels and cancer among outpatients from Germany.

**Methods:**

61,936 patients with available blood lipid values were identified from the IQVIA Disease Analyzer database and followed up between 2005 and 2019. Multivariable logistic regression models were used to study the association between lipid values and cancer.

**Results:**

The probability of cancer was significantly lower among patients with elevated total cholesterol concentrations and higher in patients with decreased HDL serum levels. In contrast, serum concentrations of LDL and triglycerides had no impact on cancer risk. In cancer site-stratified analyses, we observed a trend towards higher rates of cancers from digestive organs, breast, skin cancer, urinary tract and cancers from lymphoid and hematopoietic tissue in patients with HDL values < 35 mg/dl, while a negative association between total cholesterol > 250 mg/dl and respiratory organ as well as urinary tract cancers was observed.

**Conclusion:**

Our data strongly support the hypothesis that serum-specific lipid profiles are positively associated with cancer.

## Introduction

Changes in lifestyle factors including unhealthy diet, harmful use of alcohol, lack of physical activity, excess weight and aging of population determine the increased prevalence of high blood cholesterol and dyslipidemia worldwide (Chawla et al. 2020). In 2008, the global prevalence of elevated plasma cholesterol levels among adults was 39% (37% for males and 40% for females) (Pedersen et al. 2020). Total cholesterol serum concentration, plasma lipid profile levels (low high-density lipoprotein (HDL) and/or raised triglyceride (TG)) are key risk factors for manifold diseases. There is increasing evidence suggesting that blood lipids regulate innate and adaptive immune responses and may have anti-oxidative, anti-apoptotic, and anti-inflammatory properties (Kuvin and Karas 2003; Yvan-Charvet et al. 2010; Catapano et al. 2014), potentially promoting development of cancer (Hohneck et al. 2021). The influence of cholesterol and dyslipidemia on cancer risk has been an area of investigation for a long time. Recently, many studies and meta-analyses have analyzed alterations in blood lipids as etiologic factors for the development and progression of certain types of cancer with at least partially conflicting results. Indeed, total cholesterol levels as well as variations in HDL cholesterol levels has been associated with risks of, e.g. lung, endometrial, and colorectal cancer; however, results have been conflicting (Jafri et al. 2010; Chandler et al. 2016; Nderitu et al. 2017; Nowak and Ärnlöv 2018; Hohneck et al. 2021). In light of this controversy, the aim of this study was to provide epidemiologic evidence for the association between concentrations and profiles of serum lipids and cancer risk. We, therefore, used the Disease Analyzer database (IQVIA), which compiles drug prescriptions, diagnoses, and basic medical and demographic data obtained directly from general practitioners and specialists in Germany for about 7.000.000 patients (Rathmann et al. 2018) to dissect an association between different blood lipids and most frequent cancers.

## Methods

### Database

This study was based on data from the Disease Analyzer database (IQVIA), which contains drug prescriptions, diagnoses, and basic medical and demographic data obtained directly and in anonymous format from computer systems used in the practices of general practitioners and specialists (Rathmann et al. 2018). The database covers approximately 3% of all outpatient practices in Germany. Diagnoses (according to International Classification of Diseases, 10th revision [ICD-10]), prescriptions (according to Anatomical Therapeutic Chemical [ATC] Classification system), and the quality of reported data are being monitored by IQVIA. In Germany, the sampling methods used to select physicians' practices are appropriate for obtaining a representative database of general and specialized practices. It has previously been shown that the panel of practices included in the Disease Analyzer database is representative of general and specialized practices in Germany (Rathmann et al. 2018). For example, Rathmann et al. could show a good agreement of the incidence or prevalence of cancer diagnoses between the outpatient DA database with German reference data (Rathmann et al. 2018). Finally, this database has already been used in previous studies focusing on cancer (Huber et al. 2020; Jacob et al. 2021).

### Study population

**Fig. 1:**
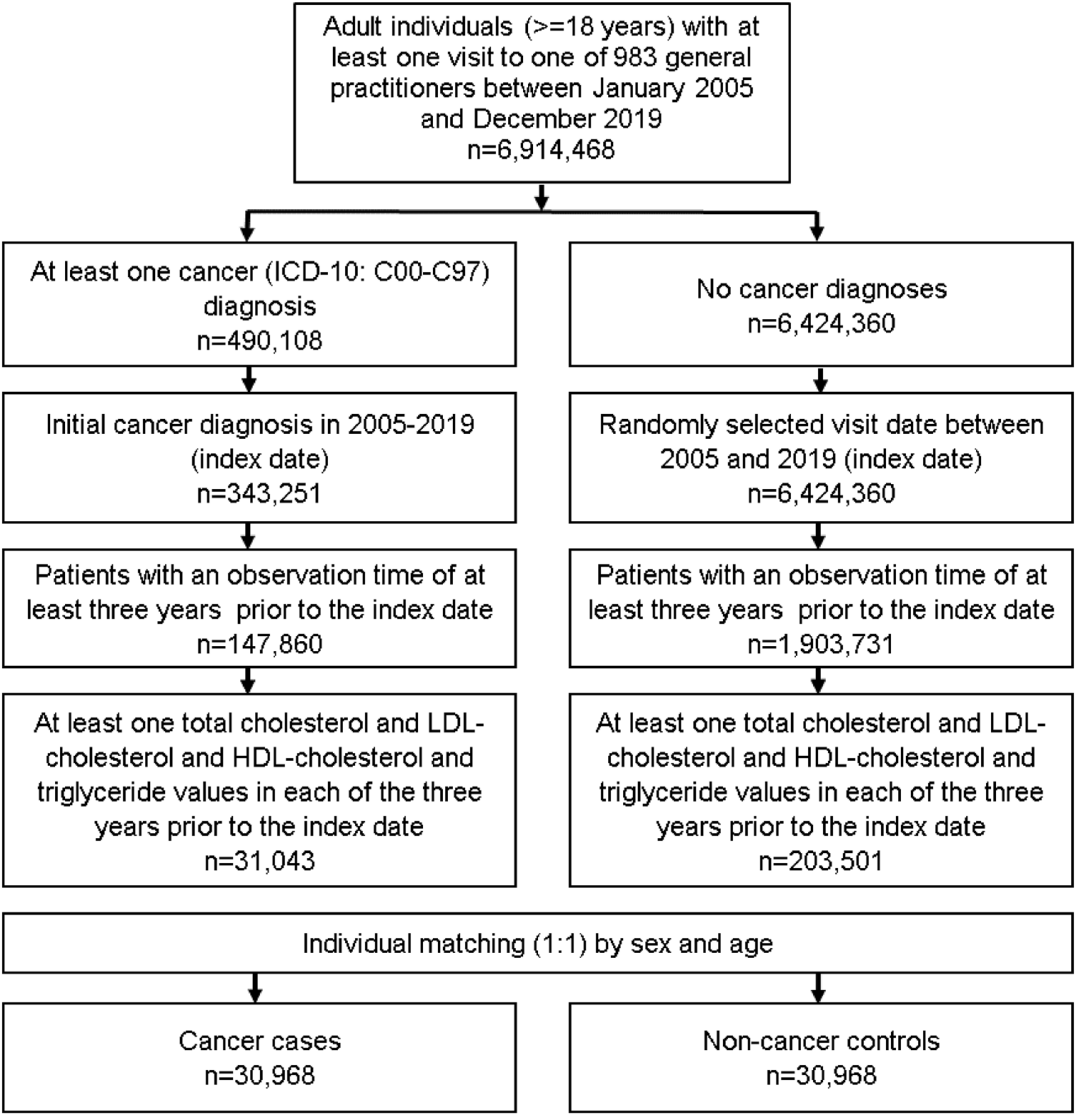
Selection of study patients

This nestle case–control study included adult patients (≥ 18 years) with an initial cancer diagnosis (ICD-10: C00-C97) in 1274 general practices in Germany between January 2005 and December 2019 (index date; Fig. 1). Further inclusion criteria were an observation time of at least 3 years prior to the index date as well as at least one total cholesterol and LDLcholesterol and HDL cholesterol and triglyceride values in each of the 3 years prior to the index date.

Cancer cases were matched to non-cancer controls by sex and age. For the controls, the index date was that of a randomly selected visit between January 2005 and December 2019 (Fig. 1).

### Study outcomes and statistical analyses

The main outcome of the study was the association between average total cholesterol, LDL cholesterol and HDL cholesterol and triglyceride values and the risk of cancer diagnosis. For each patient, average values for the four lab parameters were calculated for the time period of 3 years prior to the index date. These values were categorized in three groups: total cholesterol: < 200, 200–250, > 250 mg/dl, LDL cholesterol: < 100, 100–160, > 160 mg/dl, HDL cholesterol > 40, 31–35, < 35 mg/dl, triglyceride: < 150, 150–200, > 200 mg/dl. Differences in the sample characteristics between cancer cases and non-cancer controls were tested using McNemar tests for categorical variables and paired Wilcoxon tests for age.

A multivariable logistic regression model was conducted to study the association between average lipid values and cancer. The first model contained the four lipid values with each three categories additionally adjusted for obesity (ICD-10: E66) and diabetes mellitus (ICD-10: E10-E14) diagnoses. Values < 200 mg/dl in total cholesterol, < 100 mg/dl in LDL, > 40 mg/dl in HDL and < 150 mg/dl in triglyceride were considered reference groups. The second model contained the same variables as well as ever use versus never use of statins, fibrates and ezetimibe within 3 years prior to the index date. Regression models were calculated for all patients as well as stratified by sex and four age groups (age <  = 60, age 61–70, age 71–80, age > 80). Finally, these models were performed for each of the most frequent cancer sites including digestive organs (ICD-10: C15-C26), respiratory organs (ICD-10: C30-C39), breast (ICD-10: C50), prostate (ICD10: C61), skin (ICD-10: C43, C44), urinary tract (ICD-10: C64-C68), and lymphoid and hematopoietic tissue (ICD-10: C81-C96) versus matched non-cancer patients. All models were adjusted for obesity (ICD-10: E66) and diabetes mellitus (ICD-10: E10-E14) diagnoses. Due to the large number of different comparisons, a *p* value of < 0.001 was considered statistically significant. Analyses were carried out using SAS version 9.4 (SAS Institute, Cary, USA).

## Results

### Basic characteristics of the study sample

The present study included 30,968 patients with cancer as well as 30,968 non-cancer controls who were matched for sex and age. The basic characteristics of study patients are displayed in Table 1. The mean age [SD] of the study cohort was 71.5 [11.2] years; 46.3% were women. Diabetes and obesity diagnoses were slightly more frequent in cancer patients compared to controls. On average, each patient had 11 total cholesterol, 9 LDL, 9 HDL cholesterol and 10 triglyceride values documented prior to the index date.

**Table 1:** Age and sex structure of the study sample (after 1:1 matching by sex and age)

Variable	Proportion affected among patients with cancer (%) *N* = 30,968	Proportion affected among patients without cancer (%) *N* = 30,968	*p* value
Age (mean, SD)	71.5 (11.2)	71.5 (11.2)	1.000
Age < = 60	16.9	16.9	1.000
Age 61–70	24.4	24.4	
Age 71–80	37.1	37.1	
Age > 80	21.6	21.6	
Women	46.3	46.3	1.000
Men	53.7	53.7	
Diabetes diagnosis	45.2	41.9	< 0.001
Obesity diagnosis	18.2	16.5	< 0.001
Number of documented lab values prior to the index date			
Total cholesterol	10.7 (8.8)	10.7 (8.8)	0.950
LDL cholesterol	9.0 (7.6)	8.9 (7.6)	0.136
HDL cholesterol	9.0 (7.7)	9.0 (7.7)	0.842
Triglyceride	9.8 (8.2)	9.9 (8.5)	0.462

Proportions of patients in % given, unless otherwise indicated

*SD* standard deviation

### Low HDL cholesterol levels are positively associated with cancer

In logistic regression analysis, HDL values < 35 mg/dl were significantly associated with an increased odd of cancer (OR: 1.22, 95% CI 1.13–1.31, Table 2) as compared to HDL values > 40 mg/dl. Moreover, higher total cholesterol values were negatively associated with cancer (OR: 0.93, 95% CI 0.89–0.97 for 200–250 mg/dl and OR: 0.89, 95% CI 0.82–0.95 for > 250 mg/dl, Table 2) as compared to total cholesterol levels < 200 mg/dl. In contrast, no significant association was observed for LDL cholesterol or triglyceride levels and cancer (Table 2). In a second model, adjusted for lipid lowering therapy, these associations were similar. Lipid lowering therapy was not significantly associated with higher or lower odd of cancer (OR: 1.07, 95% CI 0.98–1.16).

**Table 2:** Association between various total, LDL and HDL cholesterol, triglyceride values and cancer (multivariable logistic regression models)

Lab value	Proportion among patients with cancer	Proportion among patients without cancer	OR (95% CI)	*p* value
Total cholesterol (mg/dl)				
< 200	43.9	40.9	Reference	
200–250	44.8	46.9	0.93 (0.89–0.97)	< 0.001
> 250	11.3	12.2	0.89 (0.82–0.95)	< 0.001
LDL cholesterol (mg/dl)				
< 100	20.5	18.6	Reference	
100–160	63.0	64.0	0.97 (0.93–1.02)	0.212
> 160	16.6	17.4	1.02 (0.94–1.09)	0.688
HDL cholesterol (mg/dl)				
> 40	84.9	86.6	Reference	
31–35	8.6	8.2	1.02 (0.96–1.09)	0.461
< 35	6.5	5.3	1.22 (1.13–1.31)	< 0.001
Triglyceride (mg/dl)				
< 150	60.6	61.0	Reference	
150–200	21.4	21.6	0.98 (0.94–1.02)	0.231
> 200	18.0	17.4	0.99 (0.94–1.04)	0.625

*Adjusted for diabetes, obesity and each of the lipid values (i.e. association between triglyceride and cancer was adjusted for LDL, HDL and total cholesterol, diabetes and obesity)

### Age- and sex stratified analyses of cancer risk

In a next step, we aimed at evaluating potential age- or sex-related associations between lipid profiles and cancer. Here, the positive association between HDL cholesterol < 35 mg/dl compared to HDL > 40 mg/dl and cancer was much stronger in women (OR: 1.56, 95% CI 1.35–1.81) compared to men (OR: 1.17, 95% CI 1.08–1.27, Table 3). In age-stratified analyses, the positive association between low HDL levels and cancer was strongest in the age group < 60 years (OR: 1.43, 95% CI 1.21–1.68, Table 3). The negative association between total cholesterol levels and cancer was significant in the age group > 80 years only (OR: 0.87, 95% CI 0.80–0.95) for 200–250 mg/dl and OR: 0.74, 95% CI 0.63–0.88 for > 250 mg/dl) as compared to < 200 mg/dl (Table 3).

**Table 3:** Association between total, LDL and HDL cholesterol, triglyceride values and cancer by age and sex (logistic regression models)

Lab value	Women	Men	Age < = 60	Age 61–70	Age 71–80	Age > 80
Total cholesterol (mg/dl)						
< 200	Reference	Reference	Reference	Reference	Reference	Reference
200–250	0.91 (0.85–0.96) *p* = 0.002	0.95 (0.90–1.00) *p* = 0.046	0.99 (0.90–1.09) *p* = 0.852	0.92 (0.85–1.00) *p* = 0.056	0.93 (0.87–0.99) *p* = 0.030	**0.87 (0.80–0.95)** ***p* < 0.001**
> 250	0.88 (0.80–0.97) *p* = 0.008	0.87 (0.78–0.98) *p* = 0.022	0.96 (0.81–1.13) *p* = 0.598	0.95 (0.83–1.10) *p* = 0.492	0.88 (0.77–0.99) *p* = 0.035	**0.74 (0.63–0.88)** ***p* < 0.001**
LDL cholesterol (mg/dl)						
< 100	Reference	Reference	Reference	Reference	Reference	Reference
100–160	0.95 (0.88–1.03) *p* = 0.212	0.98 (0.92–1.04) *p* = 0.445	0.95 (0.84–1.07) *p* = 0.427	0.90 (0.81–1.00) *p* = 0.047	0.98 (0.91–1.06) *p* = 0.600	1.02 (0.93–1.12) *p* = 0.722
> 160	1.03 (0.92–1.15) *p* = 0.616	0.98 (0.88–1.07) *p* = 0.622	0.99 (0.83–1.17) *p* = 0.901	0.87 (0.75–1.01) *p* = 0.061	1.02 (0.90–1.15) *p* = 0.793	1.23 (1.05–1.45) *p* = 0.012
HDL cholesterol (mg/dl)						
> 40	Reference	Reference	Reference	Reference	Reference	Reference
31–35	1.17 (1.03–1.33) *p* = 0.015	(0.94–1.07) *p* = 0.933	1.19 (1.03–1.36) *p* = 0.017	1.03 (0.91–1.15) *p* = 0.674	0.99 (0.90–1.09) *p* = 0.856	0.93 (0.82–1.07) *p* = 0.310
< 35	**1.56 (1.35–1.81)** ***p* < 0.001**	**1.17 (1.08–1.27)** ***p* < 0.001**	**1.43 (1.21–1.68)** ***p* < 0.001**	1.25 (1.08–1.44) *p* = 0.002	1.12 (1.00–1.26) *p* = 0.061	1.15 (0.98–1.35) *p* = 0.098
Triglyceride (mg/dl)						
< 150	Reference	Reference	Reference	Reference	Reference	Reference
150–200	1.00 (0.95–1.07) *p* = 0.886	0.95 (0.90–1.00) *p* = 0.059	1.04 (0.93–1.15) *p* = 0.513	1.01 (0.94–1.10) *p* = 0.743	0.96 (0.89–1.02) *p* = 0.178	0.92 (0.84–1.01) *p* = 0.070
> 200	1.06 (0.98–1.13) *p* = 0.140	0.94 (0.88–1.01) *p* = 0.051	0.97 (0.87–1.09) *p* = 0.619	0.97 (0.89–1.06) *p* = 0.473	1.01 (0.93–1.09) *p* = 0.856	0.98 (0.88–1.09) *p* = 0.723

Significant values are in bold

*Adjusted for diabetes, obesity and each of the lipid values (i.e. association between triglyceride and cancer was adjusted for LDL, HDL and total cholesterol, diabetes and obesity)

### Associations of lipid profiles and defined cancer sites

Finally, we aimed at further dissecting a potential association between different lipid profiles and various defined cancer sites. Although the comparatively small sample sizes did not allow to identify significant associations in cancer site-stratified analyses, we observed a tendency of a positive relationship between HDL value < 35 mg/dl and digestive organ cancer, breast cancer, skin cancer, urinary tract cancer and cancer of lymphoid and hematopoietic tissue (Table 4). In addition, we also observed a trend towards a negative association between total cholesterol > 250 mg/dl and respiratory organ cancer and urinary tract cancer (Table 4).

**Table 4:** Association between total, LDL and HDL cholesterol, triglyceride values and cancer by cancer sites (logistic regression models)

Lab value	Digestive organs *N* = 10,108	Respiratory organs *N* = 4958	Skin *N* = 12,568	Female breast *N* = 6132	Prostate *N* = 6710	Urinary tract *N* = 4520	Lymphoid and hematopoietic tissue *N* = 7526
Total cholesterol (mg/dl)							
< 200	Reference	Reference	Reference	Reference	Reference	Reference	Reference
200–250	0.88 (0.80–0.97) *p* = 0.013	0.87 (0.75–1.00) *p* = 0.043	0.97 (0.89–1.07) *p* = 0.552	0.90 (0.79–1.03) *p* = 0.131	1.07 (0.95–1.21) *p* = 0.290	0.91 (0.79–1.05) *p* = 0.209	0.91 (0.81–1.03) *p* = 0.129
> 250	0.89 (0.74–1.06) *p* = 0.182	0.70 (0.51–0.87) *p* = 0.003	1.08 (0.92–1.27) *p* = 0.344	0.93 (0.76–1.15) *p* = 0.528	0.96 (0.75–1.23) *p* = 0.734	0.73 (0.55–0.97) *p* = 0.031	0.85 (0.69–1.05) *p* = 0.131
LDL cholesterol (mg/dl)							
< 100	Reference	Reference	Reference	Reference	Reference	Reference	Reference
100–160	1.03 (0.92–1.16) *p* = 0.545	0.94 (0.79–1.11) *p* = 0.437	1.01 (0.91–1.13) *p* = 0.800	0.95 (0.80–1.13) *p* = 0.560	1.11 (0.97–1.27) *p* = 0.146	0.92 (0.78–1.09) *p* = 0.378	0.94 (0.82–1.08) *p* = 0.376
> 160	1.01 (0.84–1.20) *p* = 0.050	1.24 (0.96–1.60) *p* = 0.107	0.93 (0.79–1.09) *p* = 0.378	1.11 (0.88–1.40) *p* = 0.394	1.15 (0.92–1.45) *p* = 0.210	1.07 (0.82–1.41) *p* = 0.609	0.93 (0.75–1.14) *p* = 0.465
HDL cholesterol (mg/dl)							
> 40	Reference	Reference	Reference	Reference	Reference	Reference	Reference
31–35	0.95 (0.82–1.10) *p* = 0.468	1.25 (1.02–1.52) *p* = 0.029	1.01 (0.88–1.17) *p* = 0.851	1.19 (0.91–1.56) *p* = 0.208	0.96 (0.82–1.12) *p* = 0.581	0.95 (0.77–1.16) *p* = 0.593	1.16 (0.98–1.36) *p* = 0.090
< 35	1.29 (1.08.1.54) *p* = 0.005	1.37 (1.08–1.73) *p* = 0.010	1.22 (1.03–1.43) *p* = 0.019	1.18 (0.87–1.61) *p* = 0.298	1.10 (0.90–1.34) *p* = 0.356	1.19 (0.93–1.51) *p* = 0.160	1.27 (1.04–1.54) *p* = 0.019
Triglyceride (mg/dl)							
< 150	Reference	Reference	Reference	Reference	Reference	Reference	Reference
150–200	0.98 (0.89–1.09) *p* = 0.718	1.02 (0.88–1.17) *p* = 0.806	0.93 (0.85–1.02) *p* = 0.135	1.04 (0.91–1.18) *p* = 0.598	0.90 (0.80–1.02) *p* = 0.093	0.95 (0.82–1.11) *p* = 0.530	0.90 (0.80–1.01) *p* = 0.080
> 200	0.96 (0.85–1.07) *p* = 0.422	1.04 (0.88–1.22) *p* = 0.669	0.91 (0.82–1.01) *p* = 0.080	1.05 (0.90–1.23) *p* = 0.506	0.81 (0.70–0.94) *p* = 0.004	1.12 (0.94–1.32) *p* = 0.198	0.99 (0.86–1.13) *p* = 0.832

*Adjusted for diabetes, obesity and each of the lipid values (i.e. association between triglyceride and cancer was adjusted for LDL, HDL and total cholesterol, diabetes and obesity)

## Discussion

In recent years, it has become increasingly obvious that cholesterol and its subtypes as well as triglycerides play an important role in the development of cancer. Both, experimental and clinical analyses have demonstrated an increased incidence of cancer in patients with aberrant blood lipid profiles but the results have nevertheless remained partly inconclusive (Pedersen et al. 2020). Using the population-based IQVIA “Disease Analyzer” database, we demonstrate that the probability of cancer is significantly lower among patients with elevated total cholesterol concentrations but higher among patients with decreased HDL serum levels. In contrast, concentrations of LDL and serum triglycerides had no significant impact on cancer risk. In cancer site stratified analyses, we found a strong trend towards higher rates of digestive organ cancer, breast cancer, skin cancer, urinary tract cancer and cancer of lymphoid and hematopoietic tissue in patients with HDL values < 35 mg/dl as well as a negative association between total cholesterol > 250 mg/dl and respiratory organ as well as urinary tract cancer.

The average total cholesterol level as well as the LDL and HDL levels of the healthy normal population vary from country to country and are furthermore age and gender dependent. There is a positive correlation between blood cholesterol levels and body mass index. Cholesterol can be acquired from diet or endogenous biosynthesis. However, levels of cholesterol depend primarily on the body's own production and only secondarily on the intake from food and some studies have established the contributions of higher dietary cholesterol to cancer risk. While our dataset does not feature data on dietary patients´ habits, our results on elevated cancer risk in patients with lower total cholesterol levels and lower HDL levels appear consistent with other reports (Iso et al. 2009; Ghosh et al. 2011; Touvier et al. 2015). Moreover, analyses on individuals from two population-based cohorts (the Copenhagen General Population Study (2003–2015, *n* = 107 341), and the Copenhagen City Heart Study (1991–1994, *n* = 9387)) revealed a significantly higher cancer incidence in patients with low HDL levels. Of note, the effect was most pronounced for hematological and nervous system cancer, and to a minor extent for breast and respiratory cancer (Pedersen et al. 2020).

The pathophysiological mechanisms linking specific changes in serum lipids to higher cancer incidences are complex and only partially understood. Cancer cells have a strong affinity for sterols/lipids and lipid metabolism has been identified as a critical factor in cancer signaling (Gorin et al. 2012; Cruz et al. 2013). As an example, excessive production of lipogenic enzymes has been observed in several cancers (Nagahashi et al. 2012) and is linked with cancer severity and reoccurrence (Mashima et al. 2009; Uddin et al. 2009). Also, increased signaling activity of a combination of steroid hormone receptors and growth factors via several complex metabolic circuits (Menendez and Lupu 2007; Oliveras et al. 2010; Bhatia et al. 2011) modulate and activate SREBP-, the principal regulatory factor of lipogenesis in cancer cells. Moreover, immunomodulatory, anti-oxidative, anti-apoptotic, and anti-inflammatory properties were postulated for HDL, which might influence proliferative and inflammatory pathways in cancer development (Onwuka et al. 2020). In line, decreased HDL levels have been associated with increased levels of pro-inflammatory cytokines, including tumor necrosis factor-alpha and interleukin-6 (Haddy et al. 2003).

Our study was limited by some aspects, which are mainly related to the study design and methods and cannot be avoided (Labenz et al. 2020a). In brief, all diagnoses are coded using ICD-10 codes, which potentially leads to a misclassification and undercoding of certain diagnoses. Moreover, lab values were not available for all patients (Table 1). However, the IQVIA Disease Analyzer database that was used for the analyses of this study has been extensively published (e.g. Huber et al. 2020; Labenz et al. 2020b; Jacob et al. 2021)) and has proven its validity (Rathmann et al. 2018). Moreover, data on the socioeconomic status (e.g., education and income of patients) as well as lifestyle-related risk factors (e.g., smoking, alcohol consumption, and physical activity) are also lacking. In addition, we cannot exclude a selection bias in our study for those with diagnosis of hypercholesterinemia, meaning that patients who have an established diagnosis of hypercholesterinemia may be more likely to be examined for cancer. Although we identified over 30,000 cancer patients, subgroup analyses of individual cancer sites (e.g., colorectal cancer) were not feasible due to small samples sizes. We therefore grouped different tumor entities with similar pathomechanisms (e.g., digestive or respiratory organs), which might be associated with a presentation bias. Finally, no information is recorded why blood lipid values were taken from the patients. It seems likely that it will have been a combination of general check-up and symptom related examinations. Thus, exposure assessment was not standardized, possibly introducing an information bias and confounding by indication.

In summary, we present data from a large German primary care provider database showing that elevated levels of total cholesterol are negatively associated and lower levels of HDL cholesterol are positively associated with cancer, irrespective of diabetes, obesity, age and sex. Thus, along with previous data, our study that was based on > 60,000 patients suggests that the clinical management of patients with lipid disorders should include a careful and structured workup of cancer to improve long-term outcomes in these patients. As an example, all of these patients might be presented in a “metabolic board” and discussed with dedicated hepatologists and oncologists to recognize cancer at the earliest possible time point.
